# A nap consolidates generalized perceptual learning

**DOI:** 10.3389/frsle.2023.1168511

**Published:** 2023-09-25

**Authors:** Katherine S. Reis, Shannon Heald, Sophia Uddin, Kimberly M. Fenn, Howard C. Nusbaum

**Affiliations:** ^1^Department of Psychology, The University of Chicago, Chicago, IL, United States; ^2^Department of Otorhinolaryngology, University of Maryland School of Medicine, Baltimore, MD, United States; ^3^Department of Psychology, Michigan State University, East Lansing, MI, United States

**Keywords:** sleep, nap, learning, consolidation, perception

## Abstract

Previous research has demonstrated that a night's sleep can consolidate rote and generalized perceptual learning. Over a waking retention period following training, performance gains from learning significantly decline, but sleep can restore performance to levels found immediately after learning. Furthermore, when sleep precedes a waking retention period following training, performance is protected against loss. Other research demonstrating that rote learning can be consolidated by a night's sleep has shown that a relatively brief nap can consolidate rote learning. This suggests that short periods of sleep can produce consolidation, indicating that consolidation may not require successive sleep cycles over an entire night to emerge. However, previous research has demonstrated that there can be differences in sleep-dependent consolidation for rote and generalized learning. In this study, we investigated whether an opportunity for a 90-min midday nap was sufficient to consolidate generalized perceptual learning of synthetic speech. We recruited 75 participants from the University of Chicago community (mean age of 20.83) who completed a pretest, training, and posttest in the morning on perception of synthetic speech. Training and testing in this manner are known to result in substantial generalized learning of synthetic speech. Participants then returned in the afternoon and were either given an opportunity for a 90-min nap or remained awake for 90-min. Participants were then given another posttest later that evening, never hearing the same words twice during the experiment. Results demonstrated that participants who did not nap showed significant loss of learning at the evening posttest. In contrast, individuals who napped retained what they learned, and did not show loss of learning at the evening posttest. These results are consistent with the view that an opportunity for a 90-min midday nap can consolidate generalized learning, as only individuals with consolidated learning should be able to retain what they learned despite an intervening waking retention period. This is the first demonstration that generalized skill learning is subject to sleep-dependent consolidation in short durations of sleep and does not require a full night of sleep. This work has implications for understanding the basic neural mechanisms that operate to stabilize short-term learning experiences.

## 1. Introduction

A large body of research has shown that sleep consolidates memories by stabilizing and protecting them from forgetting (Fenn et al., 2003; Walker et al., 2003; Born and Wilhelm, 2012, etc.). Sleep-dependent memory consolidation has been widely demonstrated for both declarative and procedural learning (Plihal and Born, 1997; Backhaus and Junghanns, 2006; Alger et al., 2010). Sleep aids in the ability to recall newly learned declarative memories (Plihal and Born, 1997; Gais et al., 2006; Lahl et al., 2008) and consolidates both motor (Walker et al., 2003; Brawn et al., 2010), and perceptual learning (Stickgold et al., 2000).

It is important to note that there is a difference between rote learning—creating memories of specific experiences, stimuli, actions—and generalized learning which improves performance for stimuli that have not been specifically experienced previously (see Greenspan et al., 1988). Sleep does not simply consolidate rote learning—the repetition of a fixed set of items—but also generalized skills (e.g., Fenn et al., 2003; Brawn et al., 2008). That is, sleep is beneficial not only for consolidating memories of particular stimuli or a particular action, but also for consolidating procedural knowledge that leads to generalizable improvements in performance. Perceptual learning that is generalized in nature is thought to play a critical role in perception as we rarely experience the same stimuli twice. In the context of speech, rote-memorization of acoustic signals has been argued to be an untenable model to account for performance changes in recognition performance, due to the lack of invariance that exists between the acoustic patterns of speech and the linguistic interpretation of those patterns. As such, it has been argued that listeners rely on generalized learning to uncover how best to attend to the acoustic properties of speech for a given context (Heald and Nusbaum, 2014) and there are differences in the neural mechanisms involved in rote and generalized learning (Heald et al., 2022).

Recent research has demonstrated that when participants learn to recognize low-intelligibility synthetic speech in a context where no words repeat that participants engage in a kind of procedural learning to guide how best to direct their attention to the speech, presumably by forming an abstract representation of the talker's vocal (acoustic-phonetic) space (Heald et al., 2022). In these settings, participants who are trained and tested on all novel words, never hearing the same word twice, show significant improvements in performance, gaining on average about another 20 percentage points correct from pretest to posttest (Schwab et al., 1985; Fenn et al., 2003, 2013; Heald et al., 2022). This is a robust form of learning that persists for 6 months without additional exposure (Schwab et al., 1985). Importantly, significant performance improvement is found immediately after training, but performance deteriorates over the course of a waking day. Sleep has two effects on the fate of this learning. First, after performance degradation over a waking day, sleep can restore performance to immediate post-training levels. Second, a night of sleep after training can protect against subsequent waking degradation (Fenn et al., 2013). This pattern of results has been replicated in a generalized sensorimotor task (Brawn et al., 2008), and these studies, along with others (Sidaras et al., 2009; Pace-Schott et al., 2012; Van Hedger et al., 2015; Batterink and Paller, 2017), suggest that sleep consolidates generalized learning.

The aforementioned studies evaluate performance after a full night of sleep, but due to physiological similarities between a nap and one sleep cycle in a full night's sleep, some memory consolidation may be possible from a short daytime nap. Sleep consists of four main stages that individuals cycle through over a full night of sleep (Berry et al., 2012). Notably there is evidence to suggest that all these sleep stages can be potentially achieved in a 90-min period (Carskadon and Dement, 2005), and even as short as 45 min (Backhaus and Junghanns, 2006). Given that previous work has argued that processing that occurs during sleep stages N2, N3, and REM (McDevitt et al., 2015) may be responsible for the consolidation of perceptual learning, this then raises the possibility that consolidation may be possible from an opportunity for a 90-min day-time nap. Does the minimal amount of sleep from a nap produce consolidation of learning or is a full night's sleep necessary for consolidation to accrue over sleep cycles? If a nap presents sufficient sleep to produce some consolidation, this offers a potential explanation for why a daytime nap can prevent future daytime deterioration in a visual texture discrimination learning task and improve performance above baseline levels, similar to the improvement seen after a full night of sleep (Mednick et al., 2003). Further, additional studies have demonstrated that a nap can generate a similar benefit for consolidating declarative memories compared to a full night of sleep (Mednick et al., 2003; Tucker et al., 2006; Tucker and Fishbein, 2008; Alger et al., 2010). While it has additionally been shown that a nap as short as 6 min can promote memory performance, it has been argued that the simple onset of sleep may be enough to kickstart processes associated with memory consolidation. Under this view, memory consolidation processes once triggered by the onset of sleep are thought to continue upon waking, allowing for benefits to be realized despite only 6 min of putative sleep (Lahl et al., 2008). Taken together, there is both behavioral as well as physiological evidence that the opportunity for a 90-min midday nap can consolidate generalized learning.

Despite this evidence, however, the efficacy of short-duration sleep on the consolidation of generalized learning remains an open question. In the present experiment, we tested whether an opportunity for a 90-min afternoon nap would consolidate generalized perceptual learning of synthetic speech. The synthetic speech used in this paradigm is English speech generated by a computer program using orthographic-phonetic and phonetic-acoustic rules. The acoustic-phonetic patterns differ substantially from normally produced natural American English speech. Some acoustic cues are produced by errors in the synthetic speech model implementation and thus the speech can be misleading or incorrect in comparison with English acoustic-phonetics, whereas some are consistent but show less acoustic cue covariation that is found in natural speech (Nusbaum and Pisoni, 1985). For example, the word “bit”, when pronounced by the synthesizer, might sound more like “bat”. In this regard, learning to understand this speech might be thought of as similar to learning to understand foreign-accented speech: individuals must learn new acoustic-phonetic patterns and map them onto pre-existing phonological categories. We hypothesized that an afternoon nap would consolidate this learning and reduce waking degradation of performance. Participants were trained on synthetic speech in the morning, then randomly assigned to take a short nap in the afternoon or to remain awake. All were subsequently tested in the evening after a waking retention interval.

## 2. Materials and methods

### 2.1. Participants

We recruited 75 participants from the University of Chicago community. Participants had an average mean age of 20.83 (SD = 2.63 range: 18–32, 48 female, 27 male). All were right-handed and spoke English as a primary language with no history of speech or hearing disorders. Participants were randomly assigned to nap (*n* = 39) or to remain awake for a matched period of time (*n* = 36). There were no significant age [*t*_(73)_ = −0.46, *p* = 0.65] or gender [$X_{\left(1,\;\text{N}=75\right)}^{2}$ = 0.97, *p* = 0.33] differences between the groups.

### 2.2. Stimuli

We generated 700 monosyllabic words using *rsynth*, a text-to-speech synthesizer based on a formant-synthesis engine (Klatt, 1980). Three hundred words were used for training, and the 400 remaining words were divided into four 100-word tests matched in terms of overall recognition performance. In other words, these four tests are all balanced in terms of overall recognition performance at pretest, which indicates that each of the tests are matched in terms of initial difficulty. No word was repeated in any of the tests or during training. As a result, participants could not memorize the sound of any words to aid recognition (no rote memorization) but had to learn general acoustic-phonetic characteristics of the speech in order to improve word identification. The tests were counterbalanced across participants within each condition. Stimuli were delivered through Sennheiser HD570 headphones with a mean RMS amplitude of 66.5 dB.

### 2.3. Design and procedure

All participants arrived at the lab at 09:00 for a pretest, training, and a posttest (“posttest1”). During pretest and posttest1, participants listened to 100 unique monosyllabic synthetic speech words and transcribed what they heard by typing their responses on a computer keyboard. There was no feedback given during the tests.

During training, the 300 words were presented in 6 blocks (50 words per block). During each training block, participants first heard each of the 50 words, paired with the orthographic form of the word, displayed on the computer screen. There was a 1,000-ms inter-trial interval between words. After receiving this feedback on 50 words participants were then played the words a second time and asked to identify them. During identification trials, participants had 4 s to type in a response. If they did not respond in that time, the next stimulus was presented. Words were presented randomly during both the feedback and identification phases. After finishing each block, participants were allowed to take a short break before proceeding with the rest of the training. This first session lasted ~45 min including pretest, training, and first posttest. At the conclusion of the session, all participants were told to return at 15:00, prepared to nap although not all participants did nap (wake controls). Participants were tested in groups of two.

At 15:00, both participant groups returned and were randomly assigned to either the nap or wake condition. The nap participants were given the opportunity to nap in a quiet, dark room for 90 min. Wake control participants remained awake and were either (1) allowed to leave the lab (out-of-lab control group) until 16:30 (*n* = 16) or (2) asked to remain in the lab (in-lab control group) and not perform any tasks with audio and/or use electronics (*n* = 20). In-lab control participants were permitted to do puzzles, read, or otherwise occupy themselves but were not permitted to speak or listen to speech or music (and were not permitted access to cell phones). We created two separate wake groups to ease future data collection. Our goal was to better understand whether the results vary depending on whether participants remain in the laboratory or leave, during the 90-min control interval.

Given that the groups differ in the amount of exposure to speech with the in-lab group restricted in exposure and the out-of-lab group freely interacting with other people, it is possible that natural speech use by the out-of-lab group could adversely affect learning retention more than the in-lab group that was simply awake during this time. In order to control for acoustic interference, the in-lab control participants were housed in a room adjacent to nap participants to match general environmental noise conditions. All wake control subjects were instructed to refrain from sleeping or consuming caffeine and/or alcohol.

**Figure 1:**
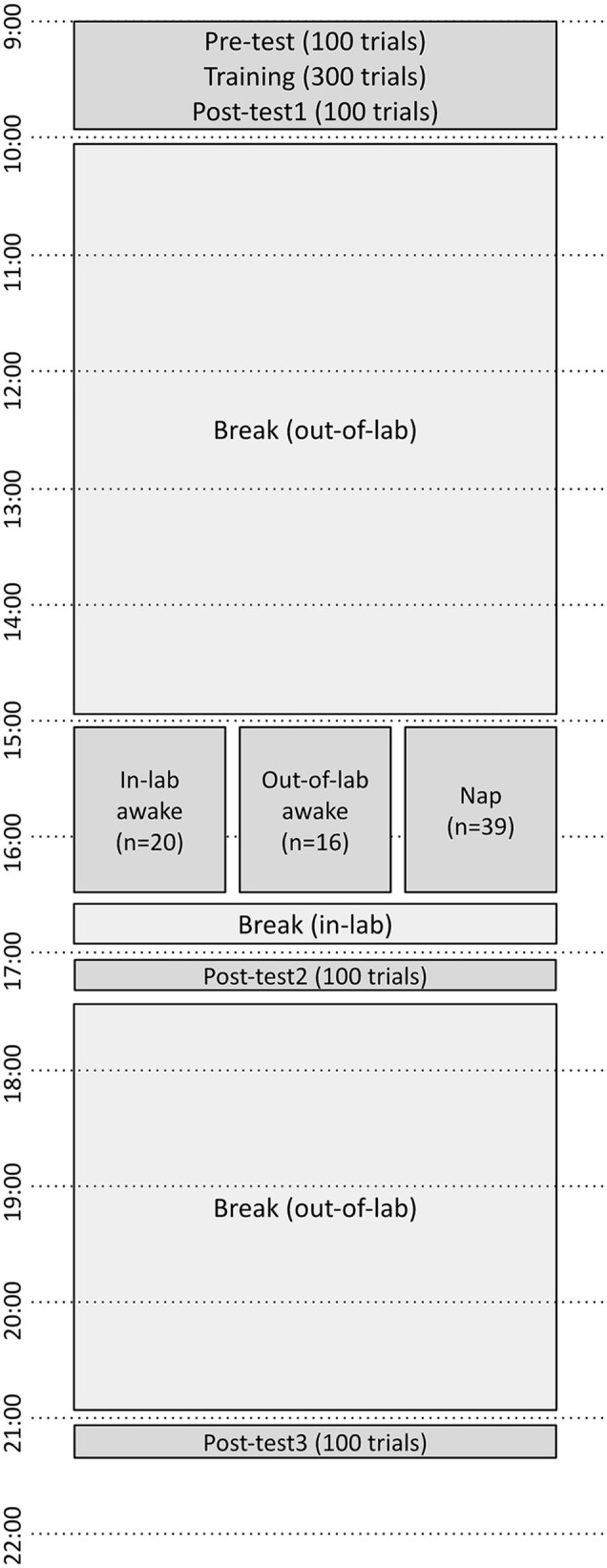
Timeline of perceptual learning tests and training over the course of the experiment. Pretest, Training, and Posttest1 began at 9:00. Subjects either napped or remained awake from 15:00 to 16:30, then performed Posttest2 at 17:00, and Posttest3 at 21:00.

At 16:30, the napping participant was awakened by the researcher, and verbal confirmation was obtained from the participant that they had slept. Control participants who did not sleep either returned to the lab (if they were assigned to the out-of-lab control) or were notified that their silent waking block was finished (if they were assigned to the in-lab control). It was confirmed that all participants assigned to the nap condition self-reported that they had napped for some portion of the nap block, and that all control participants self-reported having remained awake for the entirety of the 15:00–16:30 block. All participants then completed a second posttest (posttest2). After this, all participants were permitted to leave the lab and were instructed to refrain from napping until after their final session. All participants returned at 21:00 for the final posttest (posttest3). See Figure 1 for an outline of all study activities.

### 2.4. Data scoring

For each test, we scored accuracy based on phonetic transcription, without penalizing spelling errors. For example, if the stimulus was “tune”, the response “toon” would be scored as correct, but “tunes” would be incorrect because it included an additional phoneme. For all participant responses that did not exactly match the spelling of the stimulus, response accuracy was decided upon as a group using the International Phonetic Alphabet (IPA) using a Midwestern dialect. In the case that the spelling of a response matched a homophone found in the IPA, the spelling of the response was marked as correct. In other words, word recognition accuracy was standardized by the International Phonetic. Additionally, scorers were blind to the experimental condition to which participants were assigned.

## 3. Results

**Figure 2:**
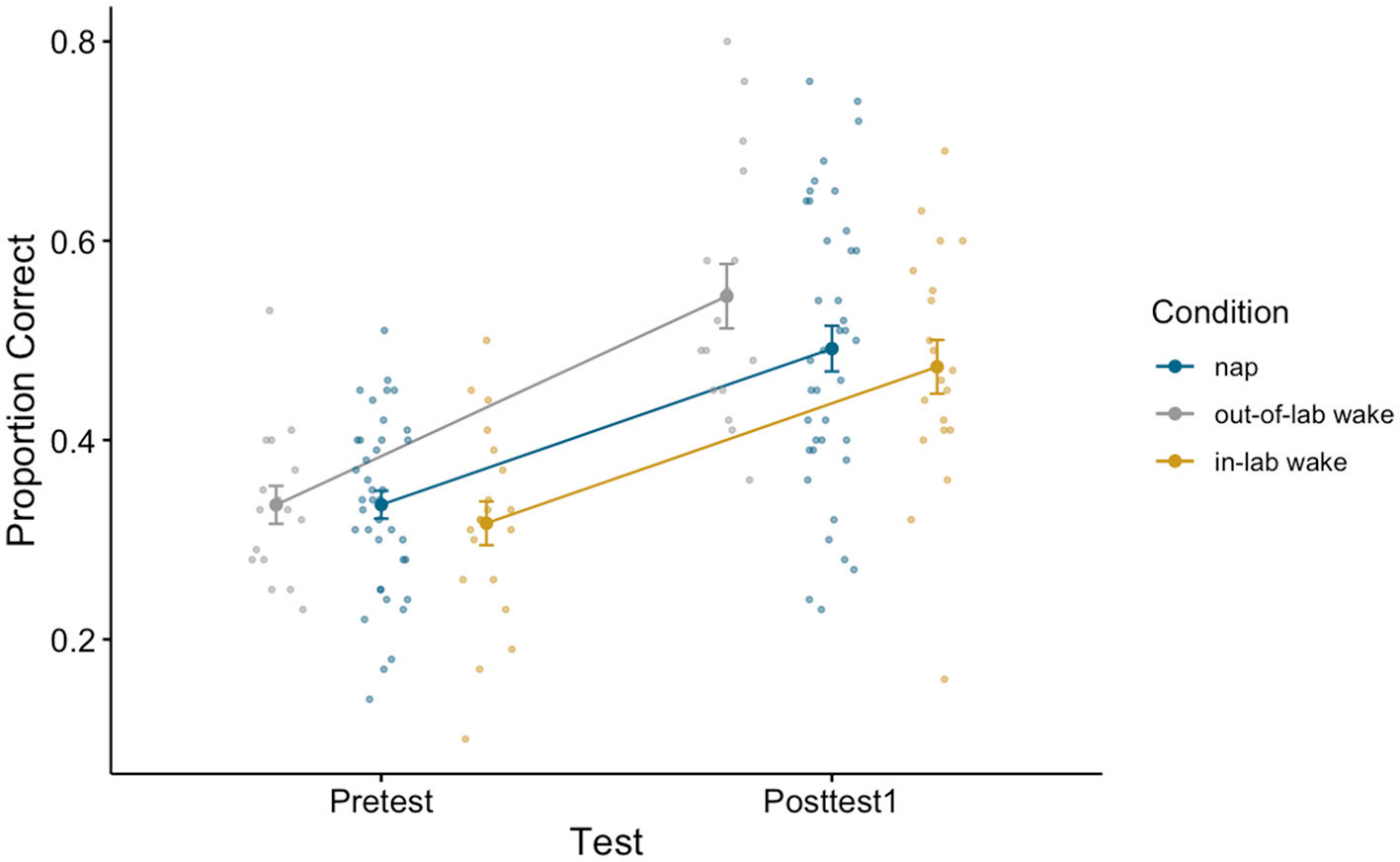
Pretest and Posttest1 performance on the perceptual learning task for all conditions. Error bars depict standard error.

To assess whether initial generalized learning did indeed occur, we compared pretest and posttest1 word recognition accuracy (proportion correct) using a 2 × 3 repeated measures ANOVA, with test (Pretest, Posttest1) as a within-subjects factor and condition (Nap, Out-of-Lab Wake, In-Lab Wake) as a between-subjects factor. Participants showed significant improvement as a result of training, as revealed by a significant main effect of test [*F*_(1,72)_ = 239.89, *p* < 0.001, η^2^ = 0.34], with an average improvement of 0.17 (pretest average: 0.33, SE: 0.01; posttest1 average: 0.50, SE: 0.02). This demonstrates that participants did indeed demonstrate generalized learning as no words repeated across the tests or training. As conditions were treated identically through posttest1, it is unsurprising that there was no significant main effect of condition [*F*_(2,72)_ = 0.82, *p* = 0.45, η^2^ = 0.01], or significant interactions between the factors [*F*_(2,72)_ = 2.11, *p* = 0.13, η^2^ = 0.01]. For estimated marginal means for each cell of the repeated measures ANOVA (see Table 1). For a plot of the proportion correct for all groups between pretest and posttest1 (see Figure 2).

**Table 1:** Estimated marginal means table for Pretest and Posttest1 performance for all conditions.

**Estimated marginal means—test** *condition					
**Condition**	**Test**	**Mean**	**SE**	**95% Confidence interval**	
				**Lower**	**Upper**
Nap	Pretest	0.34	0.01	0.31	0.36
	Posttest1	0.49	0.02	0.45	0.54
Out-of-lab wake	Pretest	0.34	0.02	0.29	0.38
	Posttest1	0.54	0.03	0.48	0.61
In-lab wake	Pretest	0.32	0.02	0.28	0.36
	Posttest1	0.47	0.03	0.41	0.53

**Figure 3:**
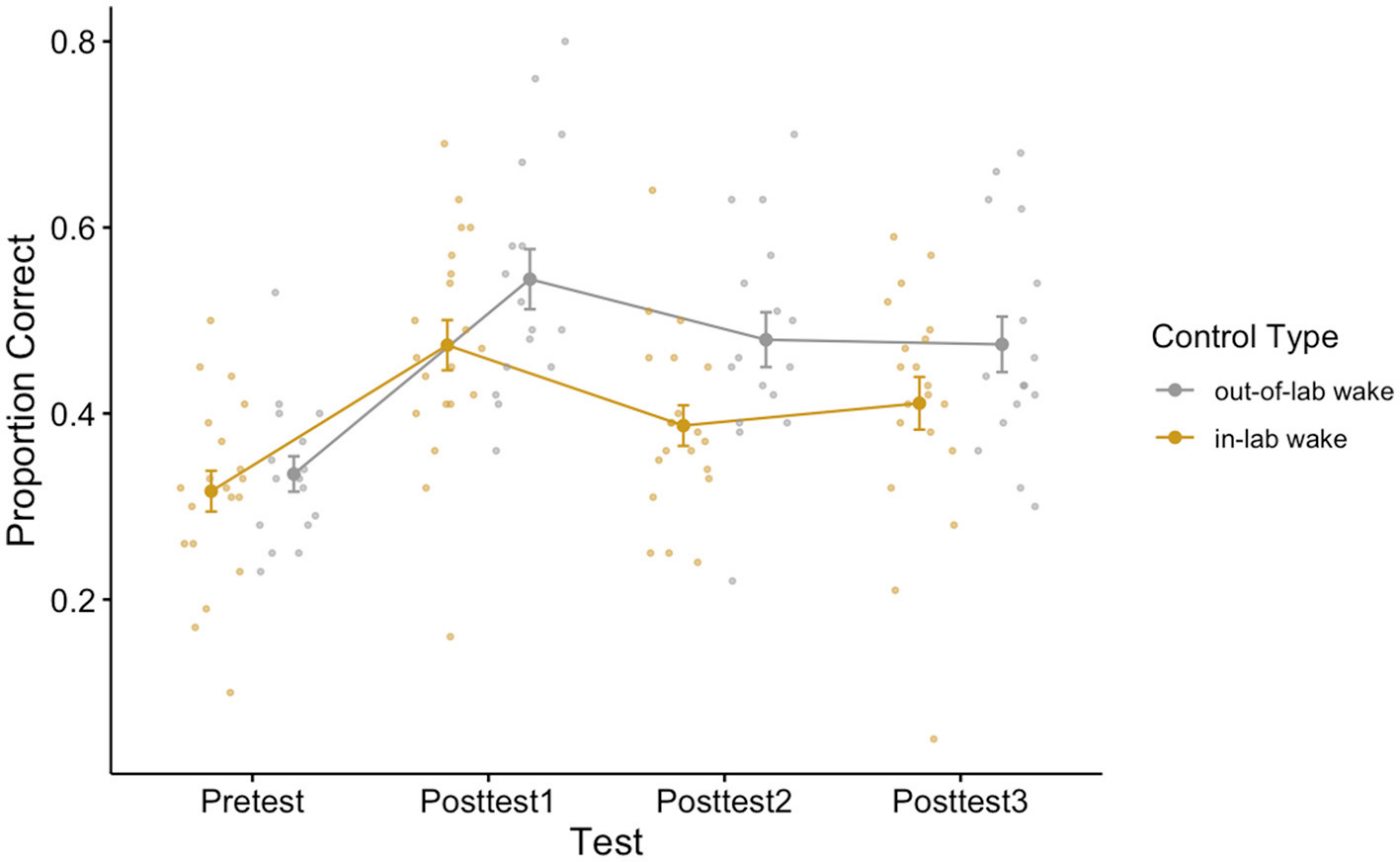
Performance of the two wake control groups across all perceptual learning tests. One control group remained in the lab during the 15:00–16:30 block (in-lab group) while another was allowed to leave the lab (out-of-lab group) during this time. Error bars depict standard error.

To see if there was a difference between our two wake control conditions across any of our tests, we additionally performed a 2 (control type: in-lab vs. out-of-lab control groups) × 4 (test block: pretest/posttests 1–3) repeated measures ANOVA. Failure to find a significant main effect of control type and failure to find an interaction effect between control type and test block, would allow us to collapse across the waking control conditions for further analyses. There was a main effect of test block [*F*_(3,102)_ = 54.30, *p* < 0.001, η^2^ = 0.25], which is to be expected as this demonstrates that participants fluctuate in performance across the day, in other words, learning and loss occurred over the course of the day. While we failed to find a significant interaction effect [*F*_(3,102)_ = 2.28, *p* = 0.08, η^2^ = 0.01], we did find a marginally significant effect of control type [*F*_(1,34)_ = 3.44, *p* = 0.07, η^2^ = 0.05] (see Figure 3 for a plot of the data associated with this analysis, as well as Table 2 for each cell of the repeated measures ANOVA). To better understand the marginally significant effect of condition we found, we conducted a *post-hoc* independent sample *t*-test to additionally assess how retained learning (posttest3–posttest1) over the course of the experiment differed between the two wake conditions. For these groups, the retained learning measure captures individual differences in post-wake retention performance relativized by individual differences in initial post-training performance. Using an independent sample *t*-test, we fail to find a difference in retained learning between the two wake groups [*t*_(34)_ = −0.30, *p* = 0.76].

**Table 2:** Estimated marginal means table for Pretest, Posttest1, Posttest2, and Posttest3 performance for both wake conditions.

**Estimated marginal means—test** ^*^control type					
**Condition**	**Test**	**Mean**	**SE**	**95% Confidence interval**	
			**Lower**	**Upper**
Out-of-lab wake	Pretest	0.34	0.02	0.29	0.38
	Posttest1	0.54	0.03	0.48	0.61
	Posttest2	0.48	0.03	0.43	0.53
	Posttest3	0.47	0.03	0.41	0.54
In-lab wake	Pretest	0.32	0.02	0.28	0.36
	Posttest1	0.47	0.03	0.42	0.53
	Posttest2	0.39	0.02	0.34	0.44
	Posttest3	0.41	0.03	0.36	0.47

Given that we could only find evidence that the control conditions acted marginally differently across the various tests, any additional variance obtained by collapsing the control conditions should only contribute to a type II error for later tests, and as such we have chosen to collapse the control conditions for the remainder of the analyses.

**Figure 4:**
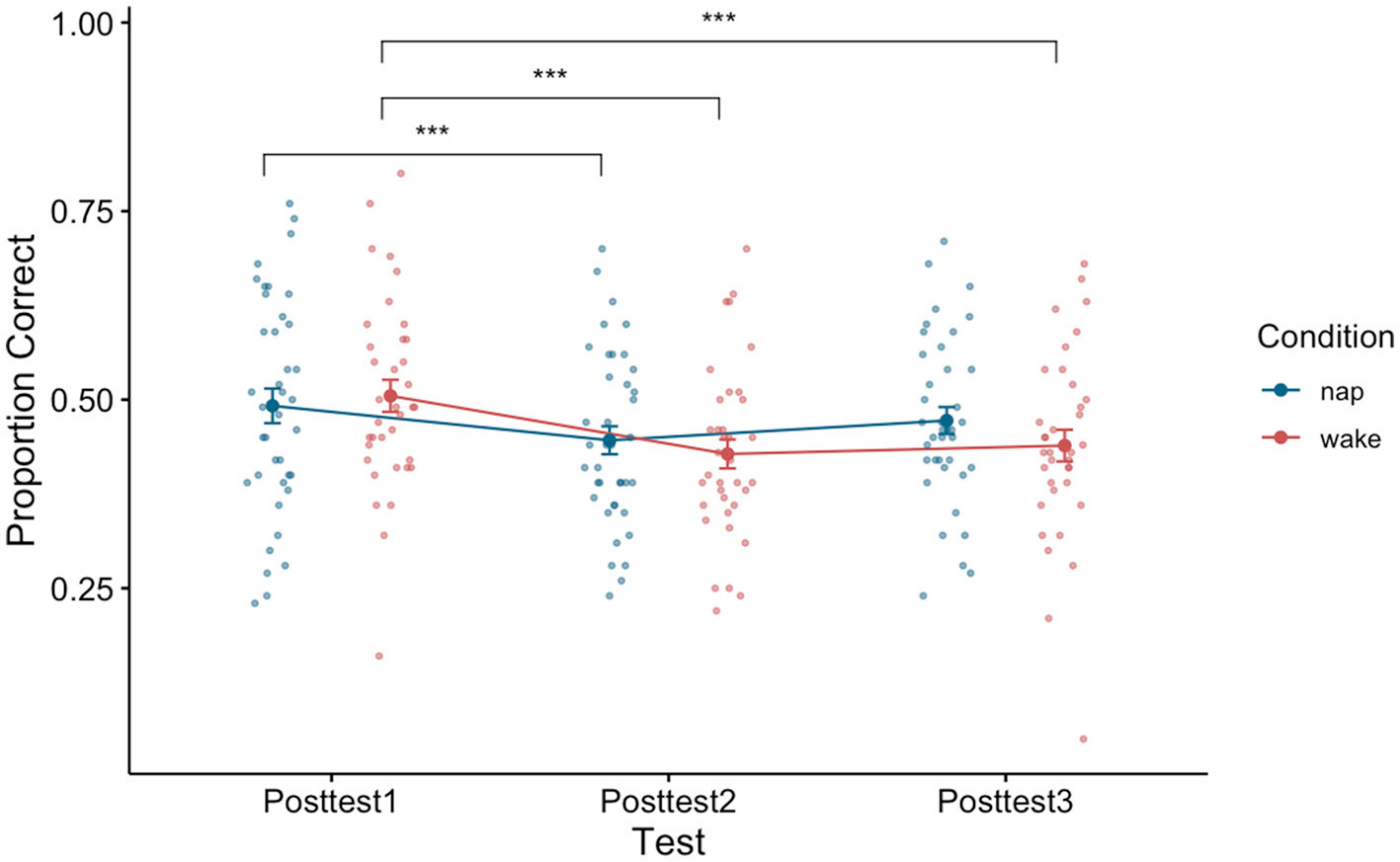
Performance between the nap and wake conditions across the three posttests. Error bars depict standard error. ***p < 0.001.

To examine the effect of napping on word recognition ability we examined test performance (proportion correct) directly after training (posttest1), directly after the sleep or wake manipulation depending on condition assignment (posttest2), and later in the day (posttest3) using a 3 × 2 repeated measure ANOVA with test (test block: Posttest1, Posttest2, and Posttest3) as a within-subjects factor and condition (condition type: Nap and Wake) as a between-subjects factor. A significant main effect of test block was found [*F*_(2,146)_ = 21.43, *p* < 0.001, η^2^ = 0.04]. A *post-hoc* Tukey test reveals that there was a significant pairwise difference between posttest1 and posttest2 (*p* < 0.001), and posttest1 and posttest3 (*p* < 0.001). By using the *post-hoc* Tukey test, we failed to find evidence that posttest2 and posttest3 (*p* = 0.13) were significantly different from one another. We additionally failed to find evidence for a main effect of condition type [*F*_(1,73)_ = 0.23, *p* = 0.63, η^2^ = 0.003]. An interaction effect between the factors [*F*_(2,146)_ = 3.03, *p* = 0.051, η^2^ = 0.01] just misses significance as well, indicating that the two condition types (Nap and Wake) performed marginally differently across the three posttests. See Figure 4 for a plot of the data associated with this analysis, as well as Table 3 for each cell of the repeated measures ANOVA.

**Table 3:** Estimated marginal means table for Pretest, Posttest1, Posttest2, and Posttest3 performance for both the condition types (nap and wake).

**Estimated marginal means—test** ^*^condition					
**Condition**	**Test**	**Mean**	**SE**	**95% Confidence interval**	
			**Lower**	**Upper**
Nap	Posttest1	0.49	0.02	0.45	0.54
	Posttest2	0.45	0.02	0.41	0.48
	Posttest3	0.47	0.02	0.43	0.51
Wake	Posttest1	0.51	0.02	0.46	0.55
	Posttest2	0.43	0.02	0.39	0.47
	Posttest3	0.44	0.02	0.40	0.48

To determine if napping influenced performance differently than being awake, we can turn to the interaction effect in this model. A marginally significant interaction effect was found [*F*_(2,146)_ = 3.03, *p* = 0.051, η^2^ = 0.01], although please note that this classification for marginal significance is due to the fact that we are reporting the associated *p*-value for this test beyond the required hundredth place. See Figure 4 for a plot of the data associated with this analysis, as well as Table 3 for each cell of the repeated measures ANOVA.

Given our a priori hypotheses and to better understand the marginally significant interaction term in the previous model, we conducted two a priori planned one-way (test block: posttest1, posttest2, and posttest3) repeated measure ANOVAs, one for the nap condition and one for the wake condition on test performance (proportion correct) directly after training (posttest1), directly after sleep (posttest2), and later in the day (posttest3). For the nap condition, we found a significant main effect of test block [*F*_(2,76)_ = 5.74, *p* = 0.01, η^2^ = 0.02]. A *post-hoc* comparison using Tukey found evidence that posttest1 and posttest2 significantly differed from one another (*p* = 0.01), although we failed to find evidence that posttest1 and posttest3 (*p* = 0.31) differed and posttest2 and posttest3 (*p* = 0.11) differed. While we would have expected sleep to restore any loss of learning, the decrease in proportion correct seen between posttest1 and posttest2 (0.05, SE: 0.01) could be due to sleep inertia. However, we see that this loss in performance is ameliorated by posttest3, as we fail to find evidence of a difference in performance between posttest1 and posttest3 (*p* = 0.31). Important to our research question, this return to initial post-training performance should only be possible if sleep consolidation has occurred. For the wake condition, we found a significant main effect of test type [*F*_(2,70)_ = 18.7, *p* < 0.001, η^2^ = 0.07]. A *post-hoc* comparison using Tukey found evidence that posttest1 and posttest2 significantly differed from one another (*p* < 0.001), posttest1 and posttest3 significantly differed from one another (*p* < 0.001), although we failed to find evidence that posttest2 and posttest3 (*p* = 0.71) differed. Here the decrease in proportion correct seen between posttest1 and posttest2 (0.08, SE: 0.01) relates to the traditional loss usually seen as a consequence of a waking retention interval. Further, we fail to find evidence that this loss in performance changes by posttest3, as we fail to find evidence of a difference in performance between posttest2 and posttest3 (*p* = 0.71). Unlike the nap condition, the wake condition participants show a significant difference between initial post-training performance and posttest3 (*p* < 0.001), which is consistent with a pattern of performance where learning has yet been consolidated.

**Figure 5:**
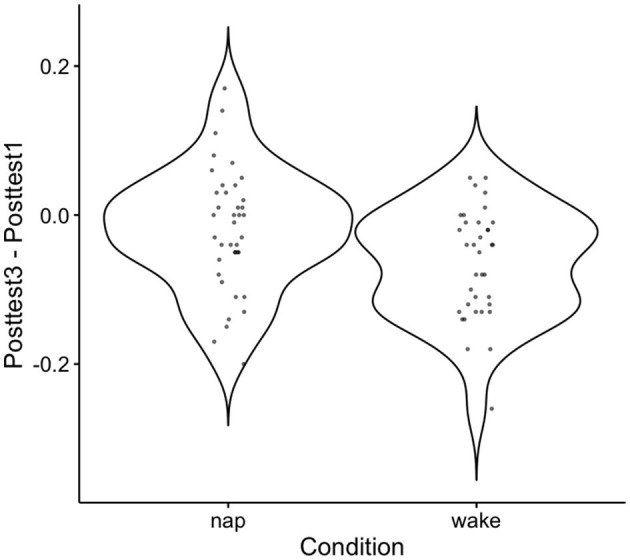
Violin plot for the distribution of retained learning (Posttest3–Posttest1) performance across subjects. The mean near zero here for nap subjects indicates an overall consolidation effect at the group level, and the dispersion around this demonstrates the observed variation found in this effect across subjects. Negative values indicate worse performance at Posttest3 than Posttest1 and positive values indicate better performance on Posttest3 than Posttest1.

While these results are consistent with the idea that the nap conditions demonstrated learning that has been consolidated, a direct statistical comparison between the groups in retained learning (posttest3–posttest1 performance) is still needed. An independent sample *t*-test on retained learning between the nap group and the wake group is indeed significant [*t*_(73)_ = 2.58, *p* = 0.01]. See Figure 5 the descriptive plot associated with this test. The results from this test demonstrate that there is a direct difference in retained learning performance as a function of the nap. Notably, we find this significant result despite having collapsed against the two waking control groups, which may have added some additional variance in performance despite a lack of statistical evidence saying that the two groups performed differently.

Furthermore, we were curious to know whether learning, and the amount of learning retained in this study was similar to previous studies where subjects slept for an entire night. In Fenn et al. (2013) a group of subjects received pretest, training, and posttest1 at 9:00, posttest2 at 21:00, and posttest3 at 9:00 the next morning after a night of sleep. Learning in this study (*M* = 0.18 ± 0.02) was similar to our nappers [*M* = 0.16 ± 0.01; *t*_(60)_ = −1.08, *p* = 0.28], and the amount of learning retained (which can be calculated by the difference in posttest3—pretest performance) is similar between Fenn et al. (2013) (*M* = 0.15 ± 0.02), and the present study [*M* = 0.14 ± 0.01; *t*_(60)_ = −0.76, *p* = 0.45]. As such, this suggests that a short bout of sleep in the middle of the day produces consolidation similar to a full night of sleep for this type of learning.

## 4. Discussion

Is the opportunity to have a 90-min nap sufficient to affect consolidation of generalized perceptual learning, and if so, is consolidation from a nap similar to consolidation from a night's sleep? Previous research (e.g., Tucker et al., 2006; Tucker and Fishbein, 2008; Alger et al., 2010) has suggested that a nap can consolidate rote learning. However, differences between rote and generalized learning, and the effect of sleep on consolidation of these forms of learning suggest that napping may not have the same effect for consolidation of generalized learning as it does for rote learning. We predicted that participants who learn to better recognize synthetic speech after training but remain awake, should show a reduction in recognition performance for novel words after the waking retention interval, even though this was a shorter retention interval than the 12 h previously investigated in Fenn et al. (2003). While both the nap and wake group showed evidence of loss between posttest1 and posttest2 (see Figure 4), we believe that this loss in the two groups arises from different sources. In the case of the nap group, the observed decrement in performance may be explained by sleep inertia, while in the case of the wake group, the loss in performance may be best explained by a waking retention interval interfering with unconsolidated learning. Sleep inertia has been found to last from anywhere from 0 min (i.e., if a fire alarm goes off) to 4 h after waking and is known to impair cognitive function (Jewett et al., 1999). This variation is thought to arise due to differences across individuals in what sleep stage they were awoken from and their sleep propensity upon going to sleep (Trotti, 2017; Hilditch and McHill, 2019). As such, it is reasonable that we may be observing effects stemming from sleep inertia at posttest2, given that this test was given only 30 min after waking.

The interpretation that sleep inertia is responsible for the decrement in performance between posttest1 and posttest 2 for the nap group is further bolstered by a return to initial post-training performance at posttest3 for those with the opportunity to nap as presumably effects of sleep inertia have dissipated by this time point. In the wake condition, however, the loss is maintained into posttest3, which is consistent with the view that experience during waking retention has had an adverse effect on unconsolidated learning. The results for those in the nap group are similar to findings observed for a full night of sleep (Fenn et al., 2003, 2013). As such we hold that the data presented here represent the first demonstration that a 90-min daytime nap opportunity—the length of an average NREM-REM sleep cycle (Carskadon and Dement, 2005)—can have the same basic effect as a full night of sleep on generalized perceptual learning. By comparison, participants who did not nap continued to show performance loss. Thus, these results demonstrate that napping does consolidate generalized perceptual learning once one takes into consideration the effects of sleep inertia. While we hold that sleep inertia is the most parsimonious source of the decrement in learning for those that napped that is found between posttest1 and posttest2, it is also possible that consolidation is not immediately made manifest during sleep but develops over waking time following sleep. In our previous human (Brawn et al., 2008, 2010) and bird (Brawn et al., 2013) studies, performance testing following sleep was not proximate to when participants woke up. Thus, evidence of consolidation always occurred after an unspecified waking interval following waking. The present study demonstrates that consolidation may not immediately present and may need to develop over the subsequent waking period. Further work is therefore needed to know if the decrement we see between posttest1 and posttest2 for the nap group is related to the slow accrual of sleep consolidation during waking retention or sleep inertia. To the degree that sleep consolidation does take time to accrue, it will be important to know whether this phenomenon is specific to a nap (fewer sleep cycles than in a night's sleep) or if it is a hallmark of consolidation more generally. These questions are relevant to understanding the time-course of consolidation and the nature of the mechanisms that support it.

While our experiments cannot distinguish between leading sleep consolidation hypotheses, they do suggest certain conclusions about the time-course of consolidation in models such as synaptic downscaling (Tononi and Cirelli, 2014) and Complementary Learning Systems (CLS, e.g., McClelland et al., 1995; Born and Wilhelm, 2012). For example, the synaptic homeostasis hypothesis proposes that (wakeful) learning potentiates synapses, while sleep preferentially downscales synapses irrelevant to the learned information (Tononi and Cirelli, 2014). Progressive synaptic downscaling occurs during NREM, and it is suggested (though not explicitly stated) that this takes place over the course of an entire night of sleep (Tononi and Cirelli, 2006; Bellesi et al., 2014). This prompts the question: how much synaptic downscaling is required to solidify learning? This suggests that consolidation occurs during sleep whereas the present evidence suggests that at the end of a sleep cycle (nap) consolidation has not yet occurred. Further, the present results demonstrate that for generalized synthetic speech learning, a full night of sleep is not necessary—given a typical average 90-min sleep cycle (Carskadon and Dement, 2005), the present results suggest that one sleep cycle is sufficient for consolidation of learning. In contrast, in systems consolidation models like CLS, memories are solidified during transfer from fast-learning to slower-learning memory stores (e.g., from hippocampus to cortex) (e.g., McClelland et al., 1995; Born and Wilhelm, 2012). Similarly, this model proposes that consolidation occurs during sleep cycles whereas the present results suggest that consolidation is not established until during the subsequent waking period.

The current results demonstrate that a daytime nap protects generalized learning of new acoustic-phonetic mappings, allowing participants to apply learning to *new* words. There is scant prior work on the impact of a nap on consolidating generalized perceptual learning. One study found that perceptual learning of motion direction detection in one direction generalized to a second direction (McDevitt et al., 2014). Another study investigated the efficacy of exposure therapy in arachnophobic participants and found that learning (defined as a reduction of the fear response to a novel spider) generalizes better after sleep than wake (Pace-Schott et al., 2012). Other generalization studies find that either a nap or a full night of sleep promotes insight and creativity (Wagner et al., 2004; Cai et al., 2009), enhances abstraction in a pattern sequence understanding task (Durrant et al., 2011), and assists performance of a visual categorization task governed by an implicit rule (Djonlagic et al., 2009). One difference between the present work and these other studies is that consolidation in the prior work could reflect generalization due to explicit inferences from declarative learning of specific episodes. In these prior cases, participants had explicit conscious access to the stimuli encountered during training and could have made inferences by generalizing consciously from them to the new situations. This cannot be true for the perceptual learning of synthetic speech. Due to categorical perception (e.g., Heald et al., 2017), listeners cannot access the auditory properties that underlie the linguistic categories that are perceived. Thus, this is a clear case in which a nap consolidates generalized perceptual learning when learning and consolidation of the exemplars themselves cannot account for the learning.

Our results also demonstrate that the source of interference responsible for the loss seen in the wake groups (in-lab and out-of-lab), is likely not due to auditory experience, as the pattern of loss was similar for awake participants who were exposed to speech outside the lab and those without this exposure. One possibility is that the loss of learning seen in the wake condition between posttest1 and posttest2 is due to reengagement of cognitive resources to another task. Generalized perceptual learning has been argued to be an active cognitive process that requires working memory and attentional resources (Heald and Nusbaum, 2014). Under this view interference does not stem from exposure to new auditory experiences but a change in how cognitive resources are used to maintain labile generalized perceptual learning previous to consolidation. Another possibility is that the loss of learning that we see in the wake condition between posttest1 and posttest2 arises via a decay function as a consequence of time simply lapsing after the conclusion of learning (Arthur et al., 1998). Future work is needed to understand the source of the loss, as this will be important to understand the role that consolidation is playing in its recovery.

The lack of an objective (physiological) sleep measure precludes relating specific features of sleep and the sleep cycle to consolidation from a nap. Nap subjects self-reported that they slept for some time during the 90-min sleep period, as we have done in past studies (e.g., Fenn et al., 2003, 2013; Brawn et al., 2008, 2010). Additionally, subjects did not immediately respond to a knock at the door, and only woke up at the time of lights on. However, the differences in performance between wake and nap groups suggest that the intervention of a 90-min nap opportunity on the whole is what is driving the results. Further research is needed to connect this variability to potential sources or mechanisms (e.g., differences in time spent in various stages of sleep; Nusbaum et al., 2018). In other words, future research using polysomnography to quantify the macro- (sleep staging) and micro-architecture (individual components of various stages of sleep, such as sleep spindles or K-complexes) of sleep, are imperative to understand how sleep specifically supports this type of memory consolidation.

In conclusion, the present study is the first to demonstrate that the opportunity for a 90-min nap is sufficient to consolidate generalized perceptual learning of speech. While it has been demonstrated that a full night of sleep consolidates generalized perceptual learning (e.g., Fenn et al., 2003, 2013), the potential of shorter naps to consolidate this type of learning was previously unexplored. As we found that the nap group retained almost the same amount of learning that sleep groups did in our previous research (Fenn et al., 2013), this suggests that, although the nap in our current study is much shorter than a night's sleep, there is very similar consolidation given comparable learning across the studies. This then leads to the question of whether certain micro- or macro-architecture overnight/during a nap is predictive of the amount of benefit toward auditory perceptual learning derived from a period of sleep. Prior work suggests that naps containing REM sleep preferentially aid rote procedural memory, while naps containing slow wave sleep assist rote declarative memory (Plihal and Born, 1997), however, this dichotomy is likely an oversimplification. Future experiments might examine the polysomnographic correlates of this type of sleep-dependent consolidation to better understand the mechanism underlying this effect, and to identify potential reasons that some individuals derive greater benefit during sleep.

## Data availability statement

The datasets presented in this study can be found in online repositories. The names of the repository/repositories and accession number(s) can be found below: https://osf.io/65y9x/. A nap consolidates generalized perceptual learning, https://doi.org/10.17605/OSF.IO/65Y9X.

## Author contributions

KSR and SH: analysis. KMF: data collection. KMF and HCN: conceptualization and design. All authors drafted portions of the manuscript and contributed to the article and approved the submitted version.
